# The anal pore route is efficient to infect *Amblyomma* spp. ticks with *Rickettsia rickettsii* and allows the assessment of the role played by infection control targets

**DOI:** 10.3389/fcimb.2023.1260390

**Published:** 2023-10-12

**Authors:** Marcelly Bastos Nassar, Daniel B. Pavanelo, Marcelo B. Labruna, Sirlei Daffre, Eliane Esteves, Andréa C. Fogaça

**Affiliations:** ^1^ Department of Parasitology, Institute of Biomedical Sciences, University of São Paulo, São Paulo, Brazil; ^2^ Department of Preventive Veterinary Medicine and Animal Health, School of Veterinary Medicine and Animal Science, University of São Paulo, São Paulo, Brazil

**Keywords:** tick, rickettsiae, RNA interference, immune factors, microplusin, anal pore

## Abstract

Adult *Amblyomma sculptum* and *Amblyomma aureolatum* ticks are partially refractory to *Rickettsia rickettsii* when fed on infected hosts, hindering the functional characterization of potentially protective targets in the bacterial acquisition. In the current study, we used the anal pore route to infect adult *A. sculptum* and *A. aureolatum* ticks with *R. rickettsii* and to assess the effects of the knockdown of microplusin in infection control. The anal pore route was efficient to infect both species, resulting in a prevalence of around 100% of infected ticks. Higher loads of *R. rickettsii* were detected in microplusin-silenced *A. aureolatum* in relation to the control, as previously obtained when microplusin-silenced ticks were fed on *R. rickettsii*-infected rabbits. This is the first report showing *R*. *rickettsii* infection through the anal pore in *Amblyomma* ticks, highlighting this route as a powerful tool to assess the role played by additional targets in the control of pathogens.

## 1 Introduction

*Rickettsia rickettsii* is the etiologic agent of Rocky Mountain spotted fever [known in Brazil as Brazilian spotted fever (BSF)], the most lethal human disease caused by a tick-borne pathogen (Dantas-Torres, 2007; Walker, 2007; Labruna, 2009). After transmission by the bite of an infected tick, *R. rickettsii* preferentially invades and proliferates into the host endothelial cells, causing vasculitis, whose progression can be fatal (Chen and Sexton, 2008). BSF is more prevalent in the southeast region of Brazil, where high fatality rates are recorded (Angerami et al., 2006; Dantas-Torres, 2007; Labruna et al., 2009), especially in the state of São Paulo (https://www.gov.br/saude/pt-br/assuntos/saude-de-a-a-z/f/febre-maculosa/situacao-epidemiologica/casos-confirmados-de-febre-maculosa-brasil-grandes-regioes-e-unidades-federadas-infeccao-2007-a-2023/view). The vectors of *R. rickettsii* in Brazil are *Amblyomma sculptum*, a member of the *Amblyomma cajennense* species complex, and *Amblyomma aureolatum* (Dantas-Torres, 2007; Labruna, 2009; Nava et al., 2014). Interestingly, *A. aureolatum* is much more susceptible to *R. rickettsii* than *A. sculptum* (Labruna et al., 2008; Martins et al., 2017). For example, when larvae of both tick species were exposed to acquisition-feeding on *R. rickettsii*-infected hosts, 80-100% of the resulting *A. aureolatum* nymphs became infected, in contrast to only 10-60% of the *A. sculptum* nymphs (Labruna et al., 2008). The transcriptional response of the midgut of these two tick species to an experimental infection with *R. rickettsii* is also distinct (Martins et al., 2017). While the majority of the coding sequences (CDS) of *A. sculptum*, including immune factors, are upregulated by infection, most of *A. aureolatum* CDS are downregulated (Martins et al., 2017).

Microplusin is an antimicrobial peptide (AMP) that was firstly isolated from the hemolymph of *Rhipicephalus microplus* (Fogaca et al., 2004) and *Amblyomma hebraeum* (Lai et al., 2004) ticks. Years later, this AMP was also identified in eggs and ovaries of *R. microplus* (Esteves et al., 2009). Microplusin is rich in histidine residues, chelating metallic ions and interfering with the respiration of both the Gram-positive bacterium *Micrococcus luteus* (Silva et al., 2009) and the fungus *Cryptococcus neoformans* (Silva et al., 2011). In a previous study, we showed that the knockdown of a microplusin (encoded by the CDS Ambaur-69859) from *A. aureolatum* by RNA interference (RNAi) increases the acquisition of *R. rickettsii* when adult ticks fed on infected rabbits (Martins et al., 2019). Indeed, the prevalence of infected ticks and the rickettsial load were higher in microplusin-silenced ticks than in non-silenced ticks. Besides showing that microplusin is important in controlling *R. rickettsii*, data also showed that adult ticks are less susceptible than larvae to infection by feeding on infected-hosts, with around only 17% of infected ticks in the control group (Martins et al., 2019). *Amblyomma sculptum* adults were also shown to be partially refractory to infection when allowed to feed on *R. rickettsii-*infected hosts (Soares et al., 2012). The low susceptibility of *A. aureolatum* (Martins et al., 2019) and *A. sculptum* (Soares et al., 2012) adult ticks to infection by feeding on infected hosts and the huge variation of the prevalence of *R. rickettsii*-infected nymphs and adults when larvae are fed on infected-hosts (Labruna et al., 2008) hampered the functional characterization of additional targets in the acquisition of *R. rickettsii*.

The administration of *Anaplasma phagocytophilum* (Taank et al., 2020) and *Borrelia burgdorferi* (Kariu et al., 2011) via the anal pore was previously reported to be efficient for infection of *Ixodes scapularis*, supporting that the anal pore is an alternative route to achieve controlled and effective infection of ticks. Therefore, in the current study, we established an alternative route to efficiently infect adult *A. aureolatum* and *A. sculptum* ticks by inoculation of *R. rickettsii* through the anal pore. To validate this route of infection in the characterization of potentially protective factors, the microplusin Ambaur-69859 of *A. aureolatum*, previously reported to control *R. rickettsii* (Martins et al., 2019), was selected. dsRNA for either microplusin or the green fluorescent protein (GFP), this latter used as a control, were injected into the ticks hemocoel. As a comparative, a similar experiment was carried out with a microplusin isoform of *A. sculptum* (encoded by the CDS Acaj-57400). After inoculation with *R. rickettsii* through the anal pore, ticks were allowed to feed on a naïve rabbit. The prevalence and the rickettsial load in tick midgut (MG) and salivary glands (SG) were determined and compared between groups.

## 2 Materials and methods

### 2.1 Ethics statement

All procedures involving vertebrate animals were carried out according to the Brazilian National Law number 11794 and approved by the Institutional Animal Care and Use Committee from the Institute of Biomedical Sciences (protocol number 1645250518), University of São Paulo, São Paulo, Brazil.

### 2.2 Ticks and *Rickettsia rickettsii*

Ticks were obtained from colonies of *A. sculptum* (Pedreira strain, São Paulo, Brazil) and *A. aureolatum* (Treze Tílias strain, Santa Catarina, Brazil) maintained at the Department of Preventive Veterinary Medicine and Animal Health, School of Veterinary Medicine and Animal Science, University of São Paulo, São Paulo, Brazil. Larvae and nymphs of *A. aureolatum* were fed on male Guinea pigs (*Cavia porcellus*), while adults were fed on male New Zealand rabbits (*Oryctolagus cuniculus*). All phases of *A. sculptum* were fed on male rabbits. Off-host phases were held in an incubator at 23°C or 25°C for *A. aureolatum* and *A. sculptum*, respectively, and 95% of relative humidity (RH). The inoculum of the high virulent Taiaçu strain of *R. rickettsii* was obtained as previously described (Martins et al., 2020).

### 2.3 dsRNA synthesis

The CDS of microplusins from *A. aureolatum* [CDS Ambaur-69859 (Martins et al., 2017; Martins et al., 2019)] and *A. sculptum* [CDS Acaj-57400 (Martins et al., 2017)] were selected as target for RNAi experiments. These two CDSs possess an identity of 82.9% and conserved cysteine and histidine residues (**Supplementary File 1**; **Figure S1**).

For cDNA synthesis, the RNA extracted from organs of either *A. aureolatum* or *A. sculptum* was treated with DNase and used as a template for reverse transcription (RT) with the reverse transcriptase M-MLV (both enzymes from Thermo Fisher Scientific, United States), according to the manufacturer’s protocol. The resulting cDNA and specific oligonucleotides for either CDS Ambaur-69859 or Acaj-57400 (**Supplementary File 2**; **Table S1**) were used in polymerase chain reaction (PCR) for amplification of specific products. As a control, specific oligonucleotides (**Supplementary File 2**; **Table S1**) and a plasmid (VR-2001-TOPO) containing a fragment of GFP coding gene were used to obtain an amplicon for GFP. Amplicons were purified using PCR Purification GeneJet™ kit (Thermo Fisher Scientific, United States) and used as template for dsRNA synthesis, according to the instructions of theT7 RiboMAX™ Express RNAi System (Promega, United States). dsRNA concentration was quantified in a spectrophotometer (Nanodrop 1000, Thermo Fisher Scientific).

### 2.4 Tick microinjection, feeding and organ collection

Unfed and noninfected adult females were affixed onto a double-sided tape and 10^11^ molecules of the dsRNAs of microplusin Ambaur-69859 (ds69859 – from *A. aureolatum*), Acaj-57400 (ds57400 – from *A. sculptum*) or GFP (dsGFP – control), suspended in 69 nL of water, were injected into the tick hemocoel using a Nanoject II equipment (Drummond, United States), as previously described (Martins et al., 2019; Nassar et al., 2023). Twenty-five biological replicates (i.e., 25 unfed females) for each group were used. After 24 h in an incubator at 25°C and 95% RH, the ticks were injected with 10^5^ genomic equivalents of *R. rickettsii* into the anal pore, also using a Nanoject II equipment. Following an additional 5 h period, ticks were allowed to feed on naïve rabbits (one rabbit for adults of each tick species for seven days). The MG and salivary glands SG of fed ticks were collected as previously described (Galletti et al., 2013). The rectal temperature of all rabbits was monitored daily for 15 days.

### 2.5 Nucleic acid extraction and cDNA synthesis

Genomic DNA (gDNA) and total RNA were simultaneously extracted from tick MG and SG using the BlackPREP Tick DNA/RNA Kit (Analytik Jena AG, Jena, Germany), following the manufacturer’s instructions. Five hundred nanograms of total RNA were treated with RQ1 RNase-free DNase (Promega, United States) and used as a template in cDNA synthesis with the M-MLV Reverse Transcriptase (Thermo Fisher Scientific, United States), according to the manufacturer’ specifications.

### 2.6 *Rickettsia rickettsii* quantification

The gDNA extracted from the MG and SG of both tick species was used as a template to quantify the genomic equivalents of *R. rickettsii* by quantitative polymerase chain reaction (qPCR). Reactions were performed using specific primers and a hydrolysis probe for the citrate synthase gene (*gltA*) of *Rickettsia* genus, as previously described (Galletti et al., 2013).

### 2.7 Quantitative PCR preceded by reverse transcription

The specific primers for microplusin isoforms (**Supplementary File 2**; **Table S1**) and the cDNA obtained as described above were used to determine gene silencing by real-time quantitative PCR preceded by reverse transcription (RT-qPCR). Reactions were performed in a StepOne™ Plus System using SYBR Green PCR Master Mix (equipment and reagent from Thermo Fisher Scientific, United States), as previously described (Martins et al., 2019). The gene expression of the ribosomal protein S3a was used as a reference (**Supplementary File 2**; **Table S1**). The relative mRNA levels (fold-change) of microplusin in either the ds69859 or the ds57400 groups in relation to that in the dsGFP group were calculated by the 2^-ΔΔCt^ method, according to the interpretation proposed by Livak and Schmittgen (2001). The percentage of gene silencing was obtained considering the microplusin expression level in the control (dsGFP) as 100%. Eight biological replicates (MG or SG of the same specimen) were analyzed.

### 2.8 Statistical analysis

The statistic differences between groups were determined by the Mann–Whitney test using GraphPad Prism (GraphPad Software Inc., version 8.0) and considered significant when *P* < 0.05. In RT-qPCR experiments, outliers were identified by the ROUT method and excluded from the analysis.

## 3 Results

### 3.1 Silencing of *A. aureolatum* microplusin by RNAi and infection through the anal pore

**Figure 1:**
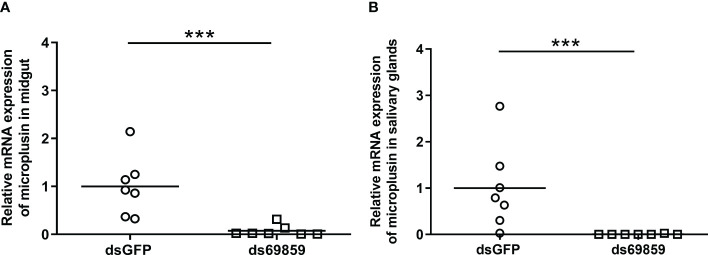
Relative expression of microplusin in *A. aureolatum* midgut **(A)** and salivary glands **(B)** of ticks injected with either ds69589 or dsGFP (control) by RT-qPCR. The percentage of microplusin gene silencing was calculated considering the expression level in control (dsGFP) as 100%. Horizontal lines represent the mean of seven ticks for each group. (***P< 0.001; Mann–Whitney test).

**Figure 2:**
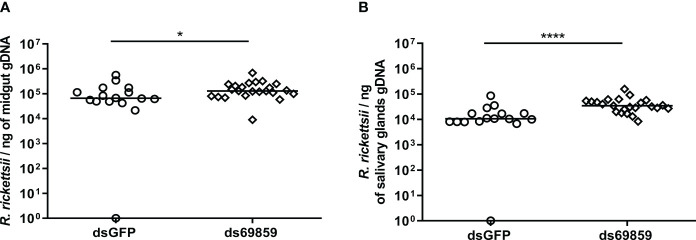
*R. rickettsii* quantification in *A. aureolatum* midgut **(A)** and salivary glands **(B)** after microplusin silencing and anal pore infection. The number of genome equivalents of *Rickettsia* was determined by qPCR using specific primers and a hydrolysis probe for the single-copy gene *gltA* of *Rickettsia* sp. Horizontal lines represent the median of all organs for each group. (*P < 0.05; ****P< 0.0001; Mann-Whitney test).

A significant reduction of microplusin mRNA levels was observed in both the MG and SG of microplusin-silenced ticks (ds69859) in relation to the control (dsGFP), with a silencing of 96% and 92% (**Figures 1A, B**), respectively. All ticks, except one specimen in dsGFP group, were positively infected by *R. rickettsii* (**Figure 2**). In addition, the rickettsial load was significantly higher in both the MG (**Figure 2A**) and SG (**Figure 2B**) of ticks from the ds69859 group compared to the dsGFP group. The rabbit used as a host for tick feeding had a fever response (temperature above 40°C) after the 11^th^ day from the feeding onset (**Supplementary File 3**; **Figure S2A**).

### 3.2 Silencing of *A. sculptum* microplusin by RNAi and infection through the anal pore

**Figure 3:**
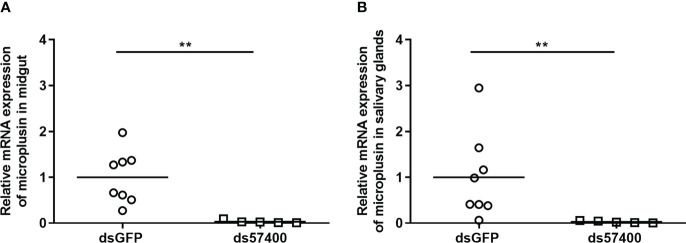
Relative expression of microplusin in *A. sculptum* midgut **(A)** and salivary glands **(B)** of ticks injected with either ds57400 or dsGFP (control) by RT-qPCR. The percentage of microplusin gene silencing was calculated considering the expression level in control (dsGFP) as 100%. Horizontal lines represent the mean of eight or five ticks (outliers were excluded) for each group. (**P< 0.01; Mann-Whitney test).

**Figure 4:**
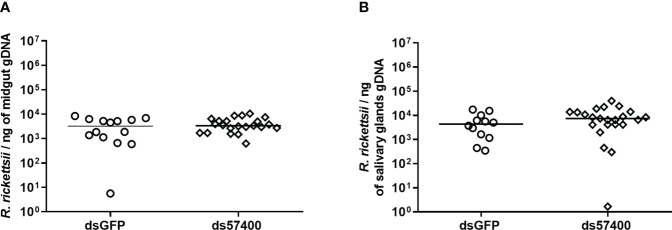
*R. rickettsii* quantification in *A. sculptum* midgut **(A)** and salivary glands **(B)** after microplusin silencing and anal pore infection. The number of genome equivalents of *Rickettsia* was determined by qPCR using specific primers and a hydrolysis probe for the single-copy gene *gltA* of *Rickettsia* spp. Horizontal lines represent the median of all organs for each group. (*P*> 0.05; Mann-Whitney test).

The efficiency of the infection of *A. sculptum* through the anal pore and the effect of the microplusin Acaj-57400 on *R. rickettsii* acquisition were determined as described for *A. aureolatum*. Microplusin mRNA levels were significatively lower in ticks of the ds57400 group than in ticks from the dsGFP group, with a reduction of 97% in both the MG and the SG (**Figure 3**). As observed for *A. aureolatum*, the prevalence of infection was around 100% in both groups. Nonetheless, there was no statistical difference in *R. rickettsii* load in MG (**Figure 4A**) or SG (**Figure 4B**) of ticks from ds57400 group in relation to the control. The rabbit used as a host for tick feeding also exhibited a fever response (temperature above 40°C) after the 11^th^ day from the feeding onset (**Supplementary File 3**; **Figure S2B**).

## 4 Discussion

A previous study of our research group has shown that the microplusin encoded by the CDS Ambaur-69859 is significantly upregulated in the MG and SG of *R. rickettsii-*infected *A. aureolatum* (Martins et al., 2019). In addition, the prevalence of infected ticks and the rickettsial load were higher in microplusin-silenced ticks than in the control group when ticks were allowed to feed on *R. rickettsii-*infected rabbits (Martins et al., 2019). In the present study, *A. aureolatum* ticks were knocked down for Ambaur-69859 microplusin, but infected through the anal pore. Ticks acquired *R. rickettsii*, confirming the efficiency of this route to infection. In addition, a higher load of *R. rickettsii* was detected in both MG and SG of microplusin-silenced ticks in relation to the control, as previously obtained when microplusin-silenced ticks were fed on *R. rickettsii*-infected rabbits (Martins et al., 2019). This result validates the anal pore route as an alternative to study the role played by additional targets in rickettsial acquisition.

The transcriptome of the midgut of *A. sculptum* infected or not with *R. rickettsii* showed that the CDS Acaj-57400, which encodes a microplusin isoform, was upregulated (1.75x) by infection (Martins et al., 2017). Considering the importance of this AMP in controlling *R. rickettsii* in *A. aureolatum* (Martins et al., 2019), the protective role of CDS Acaj-57400 was also evaluated in *A. sculptum*. Importantly, the amino acid sequence deduced from the CDS Acaj-57400 exhibit an identity higher than 80% with the microplusin encoded by the CDS Ambaur-69859 as well as conserved histidine and cysteine residues. The CDS Acaj-57400 was knocked down by RNAi followed by anal pore inoculation of *R. rickettsii*. As observed for *A. aureolatum*, ticks acquired *R. rickettsii*, showing that infection through the anal pore is also efficient in *A. sculptum*. However, there was no statistical difference in the number of bacteria in either the MG nor SG of the microplusin-silenced ticks and the control group. This result indicates that the knockdown of microplusin Acaj-57400 is not sufficient to alter the low susceptibility of *A. sculptum* to *R. rickettsii*. Indeed, it was previously shown that several immune factors are upregulated in the MG of *A. sculptum* by infection (Martins et al., 2017), suggesting that the control of *R. rickettsii* in this tick species may depend on a coordinate action of these factors.

The first organ that pathogens acquired with the blood-meal interacts with is the tick MG (Fogaca et al., 2021). The tick gut is also the first organ that bacteria inoculated through the anal pore reach, although in a different region, the hindgut. Importantly, infection through the anal pore guarantee that all ticks receive the same quantity of bacteria. Indeed, this infection route was tested and proved to be an efficient and quick method to infect *I. scapularis* nymphs with *A. phagocytophilum* and to transmit this bacterium to naïve hosts (Taank et al., 2020). The inoculation through the anal pore was also effective to infect *I. scapularis* with *B. burgdorferi*, with higher efficiency and homogeneity of the bacterial loads than the acquisition by blood feeding (Kariu et al., 2011). Here we show that this route is also efficient to infect both *A. sculptum* and *A. aureolatum* with *R. rickettsii*, being more efficient than feeding adult ticks on experimentally infected animals (Soares et al., 2012; Martins et al., 2019). As *R. rickettsii* proliferates within the host endothelial cells (Chen and Sexton, 2008), the tick ingest only few bacteria with the blood meal. Therefore, those differences in infection efficiency may be due to a higher number of rickettsiae injected into the tick anal pore than ingested with the host blood. Besides the efficiency of infection, we also observed a higher homogeneity of rickettsial loads in organs of *A. aureolatum* infected through the inoculation of *R. rickettsii* into the anal pore (around 10^4^ to 10^6^ rickettsiae) than in the organs of ticks infected by feeding on infected rabbits (around 10^2^ to 10^6^ rickettsiae) (Martins et al., 2019). The infection of ticks by inoculation of rickettsiae through the anal pore also spares the inoculation of the animals used as hosts for tick feeding.

This is the first report showing *R*. *rickettsii* infection through the anal pore in *Amblyomma* ticks. Together, the results obtained by the current study demonstrate that this route is effective to infect *Amblyomma* spp. ticks with *R. rickettsii*, even for less susceptible species, such as *A. sculptum*. The rabbits used as hosts to feed ticks exhibited fever response, indicating that *R. rickettsii* was transmitted through the tick saliva. Importantly, this alternative infection route allowed the comparative evaluation of the role played by microplusin in controlling rickettsial infection in both *A. aureolatum* and *A. sculptum* and, therefore, represents a powerful tool to study additional targets against *R. rickettsii* – and potentially other pathogens.

## Data availability statement

The original contributions presented in the study are included in the article/**Supplementary Material**. Further inquiries can be directed to the corresponding author.

## Ethics statement

The animal study was approved by Institutional Animal Care and Use Committee from the Institute of Biomedical Sciences (protocol number 1645250518), University of São Paulo, São Paulo, Brazil. The study was conducted in accordance with the local legislation and institutional requirements.

## Author contributions

MN: Conceptualization, Writing – review & editing, Data curation, Formal Analysis, Investigation, Methodology, Writing – original draft. DP: Conceptualization, Data curation, Formal Analysis, Investigation, Methodology, Writing – original draft, Writing – review & editing. ML: Writing – review & editing, Funding acquisition, Resources. SD: Funding acquisition, Resources, Writing – review & editing. EE: Writing – review & editing, Conceptualization, Data curation, Formal Analysis, Investigation, Methodology, Writing – original draft. AF: Conceptualization, Writing – review & editing, Funding acquisition, Resources, Supervision.
