# Supplementary material for: The anal pore route is efficient to infect Amblyomma spp. ticks with Rickettsia rickettsii and allows the assessment of the role played by infection control targets
Source: Front Cell Infect Microbiol. 2023 Oct 12;13:1260390. doi: 10.3389/fcimb.2023.1260390 (PMC10602902; doi:10.3389/fcimb.2023.1260390)

Supplementary File 3 – Figure S2. The rectal temperature of the rabbits used as hosts for *A. aureolatum* (**A**) and *A. sculptum* (**B**) was monitored daily for 15 days after the onset of tick feeding.

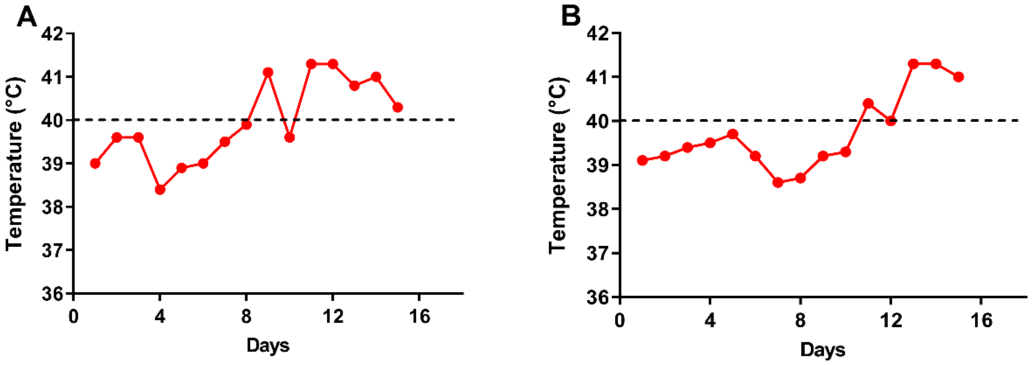

Supplement: Supplementary file 2 [file Image_2.pdf]
