# Supplementary material for: The anal pore route is efficient to infect Amblyomma spp. ticks with Rickettsia rickettsii and allows the assessment of the role played by infection control targets
Source: Front Cell Infect Microbiol. 2023 Oct 12;13:1260390. doi: 10.3389/fcimb.2023.1260390 (PMC10602902; doi:10.3389/fcimb.2023.1260390)
Supplement: Supplementary file 3 [file Table_1.pdf]

Supplementary File 2 - Table S1. Oligonucleotides used in RNAi experiments and RT-qPCR analyses.

Gene	Sense sequence (5´ → 3´)	Antisense sequence (5´ → 3´)
dsGFP	**TAATACGACTCACTATAGG**TTCACTGGAGTTGTCCCAAT	**TAATACGACTCACTATAGG**CTTGTAGTTCCCGTCATCTT
ds57400	**TAATACGACTCACTATAGG**TGACGATGCGGTGAAGAAGT	**TAATACGACTCACTATAGGG**ATGGCCATGGTCGTGACCGT
ds69859	TAATACGACTCACTATAGGGTCTTCGCCTGCTGTCTGCT	TAATACGACTCACTATAGGCTGTTGCAAATTGCCAGAGA
S3a	TACCTGCTGCGAATGTTCTG	TTCTTCCTGATGAGGCGA
Acaj-57400	GTCTTCGCCTCCTGTCTGTT	CGGTCTTCTTGCATAGCTCCAG
Ambaur-69859	TCAGTGACCACTCACCATGAA	CGGTCTTCTTGCATAGCTCCAG

*Oligonucleotides coupled to the T7 tail (bold) used for dsRNA synthesis.
