# Supplementary material for: The anal pore route is efficient to infect Amblyomma spp. ticks with Rickettsia rickettsii and allows the assessment of the role played by infection control targets
Source: Front Cell Infect Microbiol. 2023 Oct 12;13:1260390. doi: 10.3389/fcimb.2023.1260390 (PMC10602902; doi:10.3389/fcimb.2023.1260390)
Supplement: Supplementary file 1 [file Image_1.pdf]

Supplementary File 1 – Figure S1. The pairwise amino acid sequence alignment of *A. aureolatum* (Ambaur-69859) and *A. sculptum* (Acaj-57400) microplusins was performed using the EMBOSS Needle tool at the European Bioinformatics Institute (EMBL-EBI) homepage (Madeira et al., 2022). Italic letters indicate the signal peptide; bold letters highlight the conserved cysteine and histidine residues.

```
Ambaur-69859       1 MNAVFASCLFVATLVAAASAHHLELCKKTDQVLATELECIKQHIPATTNA 50
                        ||||||||..|||||||||||||||.||||||||||||||||.:||.
Acaj-57400         1 ---VFASCLFVTALVAAASAHHLELCKKNDQVLATELECIKQHIPESTNT 47

Ambaur-69859      51 AFDDAVQKLKCSDRSCAIRKMCEGNDLEGAMAKFFTTEQIKHIHDASTTC 100
                     ||||||:|::|||||||||||||||||||||||:||.|||||:|||:|||
Acaj-57400        48 AFDDAVKKVECSDRSCAIRKMCEGNDLEGAMAKYFTAEQIKHVHDAATTC 97

Ambaur-69859     101 APDAHHGHDHSHDHGHH 117
                     .||||  |||.||||||
Acaj-57400        98 DPDAH--HDHGHDHGHH 112
```


Madeira, F., Pearce, M., Tivey, A.R.N., Basutkar, P., Lee, J., Edbali, O., Madhusoodanan, N., Kolesnikov, A., Lopez, R., 2022. Search and sequence analysis tools services from EMBL-EBI in 2022. Nucleic Acids Res 50, W276-279.
